# Vascular injury in glomerulopathies: the role of the endothelium

**DOI:** 10.3389/fneph.2024.1396588

**Published:** 2024-12-23

**Authors:** Géssica Sabrine Braga Barbosa, Niels Olsen Saraiva Câmara, Felipe Lourenço Ledesma, Amaro Nunes Duarte Neto, Cristiane Bitencourt Dias

**Affiliations:** ^1^ Renal Pathophysiology Laboratory, Hospital das Clínicas, University of São Paulo School of Medicine, São Paulo, Brazil; ^2^ Discipline of Immunology, University of São Paulo School of Medicine, São Paulo, Brazil; ^3^ Department of Pathology, University of São Paulo School of Medicine, São Paulo, Brazil

**Keywords:** arteriolar hyalinosis, arteriosclerosis, glomerular endothelial cell, glomerulopathy, thrombotic microangiopathy

## Abstract

In glomerulopathies, endothelial dysfunction and the presence of histological vascular lesions such as thrombotic microangiopathy, arteriolar hyalinosis, and arteriosclerosis are related to a severe clinical course and worse renal prognosis. The endothelial cell, which naturally has anti-inflammatory and anti-thrombotic regulatory mechanisms, is particularly susceptible to damage caused by various etiologies and can become dysfunctional due to direct/indirect injury or a deficiency of protective factors. In addition, endothelial regulation and protection involve participation of the complement system, factors related to angiogenesis, the renin–angiotensin system (RAS), endothelin, the glycocalyx, the coagulation cascade, interaction between these pathways, interactions between glomerular structures (the endothelium, mesangium, podocyte, and basement membrane) and interstitial structures (tubules, arterioles and small vessels). Dysregulation of those components is also associated with the progression of renal fibrosis, since endothelial cell damage promotes endothelial-to-mesenchymal transition. Although the potential mechanisms of vascular injury have been widely described in diabetic kidney disease, hypertensive nephrosclerosis, and hemolytic uremic syndrome, they require further elucidation in other glomerulopathies. A better understanding of the pathogenesis of vascular injury in patients with glomerular diseases could contribute to the development of specific treatments for such injury.

## 1 Introduction

Vascular lesions are important findings in renal histology of glomerular diseases. The presence of thrombotic microangiopathy, arteriolar hyalinosis and arteriosclerosis can guide differential diagnoses, therapeutic options and prognosis. The pathogenesis of vascular injury can be multifactorial, but the initial process seems to be related to endothelial dysfunction resulting from endothelial injury (1).

Damage to the endothelial cells of the glomeruli, renal arterioles, and renal arteries can occur in various etiologies, such as autoimmune diseases, complement system dysregulation, preeclampsia, diabetic kidney disease and hypertensive nephrosclerosis. In primary glomerulopathies such as immunoglobulin A nephropathy (IgAN), focal segmental glomerulosclerosis (FSGS), and membranous nephropathy, vascular injury is less common, but when present they confer a worse renal prognosis (2–6). In diseases in which the mechanism of endothelial injury is better elucidated, guided therapy provides significant clinical benefit. Atypical hemolytic uremic syndrome (aHUS) provides an example of how the discovery of a dysregulation in the complement system, as the initial mechanism for the occurrence of thrombotic microangiopathy (TMA), led to the development of treatments that block components of the complement system, which have modified the trajectory of the disease (7). Other glomerular diseases dependent on complement activation, including primary membranoproliferative glomerulonephritis and C3 glomerulopathy (C3G), can also present with vascular injury, and therapies involving complement inhibition have been tested in these conditions (8–10). In preeclampsia, the discovery of an imbalance between angiogenic factors, as a central mechanism of systemic endothelial dysfunction and vascular damage, has allowed for better clarification of the diagnosis and better monitoring of pregnant women at high risk for developing that complication (11). Beyond these conditions, the mechanisms of vascular injury have been widely described mainly in diabetic kidney disease, hypertensive nephrosclerosis, and ANCA-associated vasculitis (12–14). More recently, some studies have addressed vascular injury related to lupus nephritis (LN) and IgAN, although the exact mechanisms have not yet been fully elucidated (15, 16). The presence and pathogenesis of endothelial involvement in other glomerular diseases merit further analysis.

Understanding the mechanisms of endothelial dysfunction in glomerular disease can be complex, because endothelial regulation and protection involve participation of the complement system, factors related to angiogenesis, the renin–angiotensin system (RAS), endothelin, the glycocalyx, the coagulation cascade, interaction between these pathways, interactions between glomerular structures (the endothelium, mesangium, podocyte, and basement membrane) and interstitial structures (tubules, arterioles and small vessels) (1).

The aim of this review is to describe the potential mechanisms of endothelial dysfunction and discuss the occurrence of vascular lesions in glomerulopathies.

## 2 The glomerular endothelium

Renal endothelial cells have specific characteristics highlighting the glomerular endothelium, which is highly fenestrated and covered by the rich structure of the glycocalyx. The glomerular endothelium is part of the glomerular filtration barrier, influences vascular permeability, and helps maintain podocyte morphology. The glycocalyx is a thin layer with a negative charge, composed of proteoglycans (mainly heparan sulfate and chondroitin) and glycosaminoglycans (mainly hyaluronic acid), and is essential for regulating the glomerular filtration barrier (1). The glomerular endothelium, in addition to presenting anti-inflammatory and anti-thrombotic regulatory mechanisms, including components of the complement system, also expresses (17): vasoactive factors, such as endothelin-1 (ET-1), prostacyclin, nitric oxide, and the vascular endothelial growth factor (VEGF) receptor; intercellular adhesion molecules, such as platelet endothelial cell adhesion molecule-1, intercellular adhesion molecule-1, intercellular adhesion molecule-2, and vascular adhesion cell molecule-1; and thrombotic regulators, such as von Willebrand factor, tissue factor, plasminogen activator, and plasminogen activator inhibitor-1. All these components contribute to the integrity of the endothelial cell.

## 3 Glomerular endothelial dysfunction

**Figure 1:**
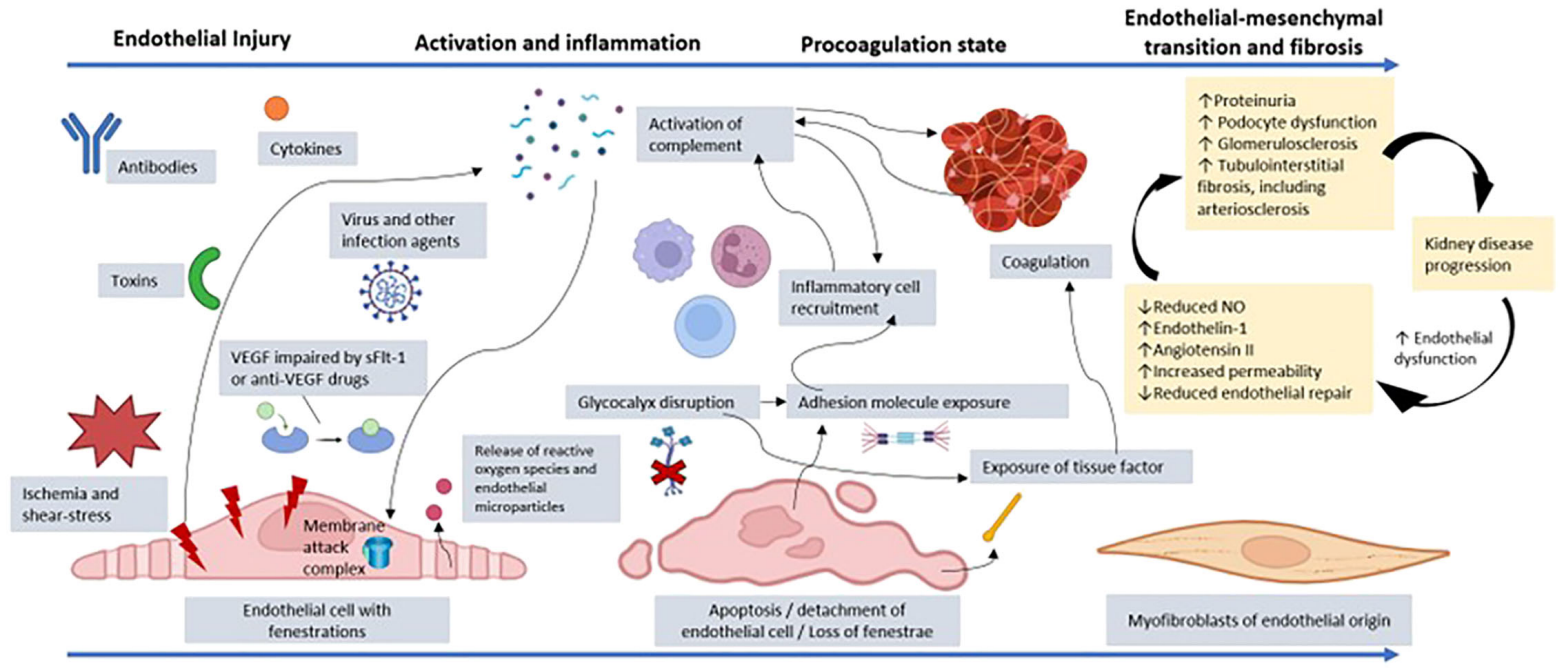
Representation of the process of endothelial dysfunction resulting from injury to a glomerular endothelial cell by toxins, cytokines, antibodies, infectious agents, VEGF depletion, ischemia, or another factor. Cellular injury promotes the release of reactive oxygen species and endothelial microparticles. There is activation of the complement system, which can culminate in the production of the membrane attack complex and endothelial lysis. In addition, anaphylatoxins from the complement system, such as C3a and C5a, promote recruitment of inflammatory cells, amplifying the inflammatory state. The disruption of the glycocalyx and the endothelial injury itself promote exposure of adhesion molecules, which increases the connection with other inflammatory mediators. There is also exposure of tissue factor with activation of prothrombotic mechanisms, which favors microthrombi affecting the microcirculation. The coagulation system is also activated through dysregulation of the complement system, which favors platelet aggregation via cytokines. Given that the insult persists, and endothelial repair is impaired, there can be loss of fenestrations and endothelial cell detachment, with reduced vascular permeability. The persistent dysfunction evolves with a change from the endothelial to the mesenchymal phenotype, contributing to renal fibrosis. Furthermore, there is increased expression of the angiotensin II and endothelin-1 receptors, which act by promoting vasoconstriction by decreasing nitric oxide (NO) production, thus increasing inflammation and fibrosis. The interaction between the endothelium, podocyte, and tubulointerstitium is dysregulated, increasing proteinuria and contributing to glomerulosclerosis and tubulointerstitial fibrosis, including impairment of arterioles and small vessels. Finally, there is accelerated progression to chronic kidney disease because uremic toxins perpetuate the state of endothelial dysfunction. VEGF, vascular endothelial growth factor; sFlt-1, soluble fms-like tyrosine kinase-1. (Created with BioRender.com).

In glomerular diseases, endothelial damage triggered by immune system dysfunction, cytokines, toxins, ischemia or deficiency of endothelial protective factors (the glycocalyx, angiogenic factors, or complement regulators) can lead to the loss of endothelial integrity and consequently endothelial dysfunction (1). This process begins with an initial activation/inflammation phase and culminates in fibrosis, which is associated with the progression of kidney disease (17), as illustrated in **Figure 1**.

## 4 Markers of endothelial dysfunction

The main markers of endothelial injury that can be the target of research to understand the pathogenesis and treatment of some glomerular diseases are described below.

### 4.1 Plasma markers

Adhesion molecules (soluble vascular adhesion cell molecule-1, soluble intercellular adhesion molecule-1, and soluble E-selectin) are found at high levels during endothelial dysfunction, as are pro-inflammatory markers (inflammatory cytokines and endocan), thrombotic mediators (thrombomodulin, von Willebrand factor, and tissue factor), markers of nitric oxide dysregulation highlighting dimethylarginine and markers of glycocalyx disruption, such as heparan sulfate (1).

Below, we explore other plasma markers of endothelial damage considered relevant for understanding the pathogenesis of glomerular diseases, vascular lesions and as targets for treatment.

#### 4.1.1 Vascular endothelial growth factor

The most important pro-angiogenic factor is VEGF. It also regulates endothelial cell function through the induction of nitric oxide, promoting vasodilation, decreased vascular tone, and reduced blood pressure. In the kidney, VEGF is located mainly in the podocyte, playing an important role in the maintenance and stability of the glomerular filtration barrier (18, 19). Situations that cause a reduction in VEGF expression are associated with endothelial and podocyte damage, leading to proteinuria, hypertension, and the TMA as a more severe presentation (18). The main scenarios associated with reduced VEGF expression are the use of anti-VEGF drugs, such as for the treatment of neoplasms, and preeclampsia, caused by a disproportionate increase in soluble fms-like tyrosine kinase-1 (produced in the process of inadequate placentation), which binds to circulating VEGF and placental growth factor, thus preventing them from interacting with their endothelial receptors (18). In recent years, many studies have also demonstrated the involvement of VEGF in the progression of chronic kidney disease and as a prognostic marker in glomerulopathies, including lupus nephritis and membranous nephropathy (19–22).

#### 4.1.2 Angiotensin II

Angiotensin II (AngII) is the main effector of the RAS, being responsible for activating several mechanisms involved in vasoconstriction, as well as pro-oxidant and inflammatory pathways that affect endothelial cell function (23). The effects of AngII activity occur through the action of AngII receptors, mainly the angiotensin type 1 receptor (AT_1_R). After binding to AT_1_R, AngII exerts vasoconstrictive effects (from the release of Ca^2+^, leading to an increase in vascular tone), together with prothrombotic, pro-oxidant, and antifibrinolytic effects, as well as stimulating the expression of pro-inflammatory, atherogenic, and fibrogenic factors (24, 25).

It is known that AT_1_R is present in various cells of the body, especially in the smooth muscle of arteries and arterioles. In the kidney, it is expressed not only in arteries and the endothelium but also in the medulla, proximal tubular epithelium (where it plays a role in sodium reabsorption), podocytes, and glomerular mesangial cells (26). Vasoconstriction of the efferent arteriole, caused by AngII, leads to increased intraglomerular pressure, resulting in proteinuria and glomerular sclerosis. In addition to the hemodynamic effect, the inflammatory stimulus is associated with progression to fibrosis and progression of kidney disease. There is increased expression of AngII and AT_1_R in the renal interstitial cells of patients with progressive glomerulopathies, mainly in those with interstitial fibrosis (27). It is known that atrophy and tubulointerstitial fibrosis are important markers of the progression of kidney disease in many glomerulopathies. The use of AngII receptor blockers and AngII-converting enzyme inhibitors has proven benefits in effectively and safely reducing the progression of renal fibrosis, improving blood pressure control, as well as cardiovascular and renal outcomes (28–30).

The association between vascular injury and interstitial fibrosis could explain the fact that glomerulopathies with ischemic vascular lesions, such as IgAN, membranous glomerulopathy, FSGS, and LN, have worse prognoses than glomerulopathies without such lesions. Increased AT_1_R expression plays a fundamental role in renal fibrogenesis, which also involves the participation of ET-1, another potent vasoconstrictor present in progressive glomerulopathies, the blockade of which reveals a renoprotective effect (31). Studies have shown that AngII acts to increase ET-1–induced vasoconstriction by increasing the expression of the ET-1 receptor and the binding between ET-1 and its receptor (32).

In addition, AngII regulates and increases the expression of transient receptor potential channel C6, a calcium-dependent channel associated with the slit diaphragm. In animal models, it has been shown that this increased activity through AT_1_R leads to cytoskeletal disruption, podocyte damage, and glomerulosclerosis (33).

#### 4.1.3 Agonist angiotensin II type 1 receptor autoantibody

**Figure 2:**
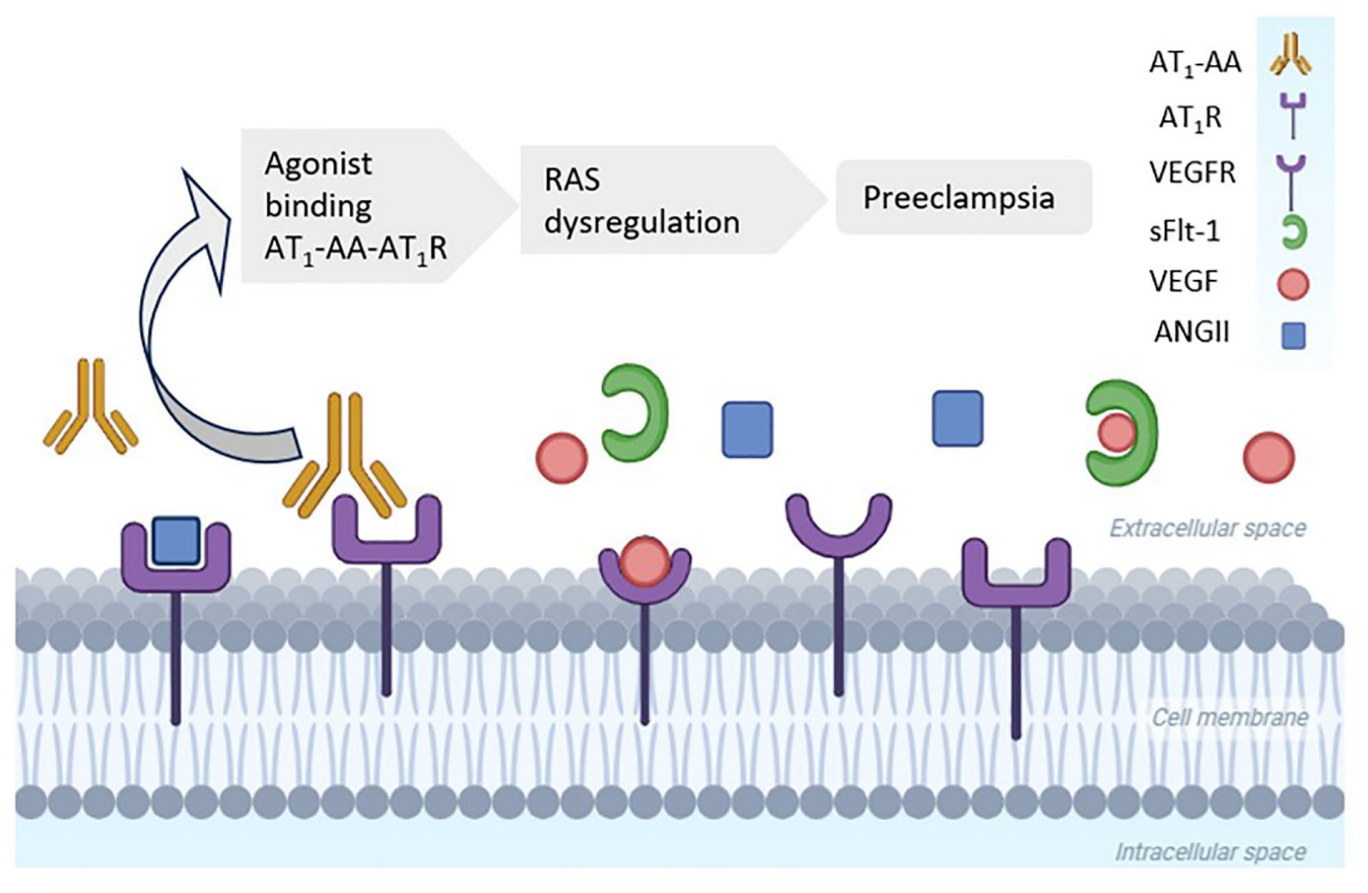
High levels of agonist angiotensin II (AngII) type 1 receptor autoantibody (AT_1_-AA) in women with preeclampsia. AT_1_-AA binds to the angiotensin type 1 receptor (AT_1_R), whereas soluble fms-like tyrosine kinase-1 (sFlt-1) blocks the vascular endothelial growth factor receptor (VEGFR). Both processes promote a hypertensive status in preeclampsia. RAS, renin–angiotensin system. (Created with BioRender.com).

In 1999, Wallukat et al. identified the previously unknown agonist angiotensin II type 1 receptor autoantibody (AT1-AA) in the circulation of pregnant women with preeclampsia (34). It is primarily a member of the immunoglobulin G_3_ antibody subclass that has an agonistic action in the AT_1_R, thus exerting vasoconstrictive effects similar to those of AngII (25). Depending on the clinical scenario, it can present as one of the other immunoglobulin G subclasses. This antibody binds with high affinity to AT_1_R, promoting permanent stimulation. It is known that renin, angiotensin, and aldosterone levels are reduced in pregnant women with preeclampsia. Activation of AT_1_R by AT_1_-AA would therefore explain the occurrence of systemic vasoconstriction and hypertension in preeclampsia, even with low levels of RAS components (25), as represented in **Figure 2**.

Various studies have corroborated the correlation between AT_1_-AA positivity and the occurrence of preeclampsia. Experimental animal models of pregnancy have shown that AT_1_-AA injection triggers the manifestation of maternal preeclampsia syndrome, with hypertension, proteinuria, and glomerular endotheliosis, the main renal histopathological finding of endothelial injury in preeclampsia (35). Subsequent studies implicated AT_1_-AA in vascular transplant rejection, malignant hypertension, glomerulopathies, scleroderma and other scenarios, as well as showing that it is associated with accelerated vascular senescence (34, 36–38).

Some studies have analyzed the role of AT_1_-AA in glomerulopathies. In a study of patients with LN, the AT_1_-AA positivity rate was 66% (39). In that study, AT_1_-AA levels were higher among the patients without immunosuppression and with other signs of disease activity such as complement consumption and high anti-DNA titers. In a subsequent study (40), the association between AT_1_-AA and vascular damage was analyzed in patients with LN. In that study, renal biopsies showed that medial layer hypertrophy and subintimal fibrosis were greater in the vessels of AT_1_-AA–positive patients than in those of AT_1_-AA–negative patients. One report described the case of an AT_1_-AA–positive patient with LN who underwent kidney transplantation and developed non-human leukocyte antigen antibody-mediated rejection, together with collapsing FSGS (41). The authors reported that the patient presented a good response to treatment with plasmapheresis, immunoglobulin, and AT_1_R blockade. It has been demonstrated that the levels of AT_1_-AA are significantly higher in patients with LN than in those with other primary glomerulopathies, such as membranous glomerulonephritis, IgAN, and FSGS (38), although no significant differences have been detected in comparison with patients with cytoplasmic or perinuclear antineutrophil cytoplasmic antibody (ANCA)-associated vasculitis. It is known that severe vascular injury, such as that provoked by TMA, is associated with worse outcomes in LN. The incidence of TMA is greater in LN with higher levels of activity, which somehow provides an immunological stimulus for vascular damage (42). The presence of AT_1_-AA is correlated with higher levels of immunological activity in patients with LN (38). Therefore, AT_1_-AA might play a central role in intense immunological stimulation and vascular damage.

#### 4.1.4 Endothelin

Although it was originally described as an endothelium-derived vasoconstrictor, ET-1 is now known to play a role in cell proliferation, water–sodium balance, acid–base balance, tissue injury, fibrosis, and the progression of kidney disease, depending on which receptor is activated (43). The two main receptors are endothelin receptor type A (through which ET-1 promotes vasoconstriction, cell proliferation, and matrix accumulation) and endothelin receptor type B (through which ET-1 promotes antiproliferative, antifibrotic, and vasodilatory effects), being the type A expressed predominantly in vascular smooth muscle cells and the type B in vascular smooth muscle cells and endothelial cells, but both of which are also found in the mesangium, podocytes and tubules (44). Plasma ET-1 is increased in conditions of inflammation and vascular damage, correlating with albuminuria, and a high plasma ET-1 level is also an independent predictor of vascular dysfunction in chronic kidney disease (43). Treatment with selective endothelin A receptor blockers has been shown to provide a significant reduction in proteinuria, making it a useful tool in the treatment of FSGS, IgAN, and chronic kidney disease, mainly in combination with an AT_1_R antagonist (45–48).

#### 4.1.5 Complement system

The complement system is a crucial part of the innate immune response, being responsible for clearing microorganisms, damaged cells, and immune complexes, as well as promoting inflammation and attacking the cell membranes of pathogens (49). The complement system can be activated through the lectin, classical, and alternative pathways leading to the generation of the C5b9, known as the membrane attack complex, as reviewed by Yoshida Y et al. (50). On the endothelial cell surface, C5b9 stimulates the secretion of von Willebrand factor, stimulates endothelial prothrombinase activity, and induces tissue factor expression. This process can involve nuclear factor kappa B, inflammatory cytokines, and culminates in an intense state of inflammation and microvascular coagulation (51).

Various proteins act as complement system regulators to prevent exacerbated activation of the complement system and protect autologous tissues, such as glomerular endothelial cells, against attack by the complement system. The main ones are membrane cofactor protein (MCP/CD46), Complement Factor H (CFH), Complement Factor I (CFI), C4b-binding protein, CR1, decay accelerating factor (DAF/CD55), and CD59 (50).

In aHUS, the dysfunction of regulatory or activating factors is associated with the pathogenesis of vascular damage, and the dosage of some components can show the complement system activation, although only genetics tests can define the constitutive complement system dysregulation (52). CFH is a fluid-phase protein, but it is also found on the cell surface, binding to glycosaminoglycans and sialic acid. In fact, CFH dysfunction is detected in aHUS and C3G, suggesting that it plays an important role in the pathogenesis of TMA in these glomerular disease (50). In some glomerulopathies, the serum level of many factors can be associated with active disease, such as C3a and C5a in ANCA-vasculitis, and soluble C5b-9 in C3G (52). MCP, CR1, CD55, and CD59 are expressed in the glomerulus and can be altered in several glomerular disorders, such as IgAN, lupus nephritis, membranous nephropathy, and primary membranoproliferative glomerulonephritis (53). More details on the mechanisms of complement-endothelial interactions in glomerulopathies are described in section 5.

#### 4.1.6 Coagulation system

Under physiological conditions, the endothelium maintains the environment in a more anticoagulant state by producing regulatory factors and through the action of the undamaged glycocalyx. The glycocalyx disruption exposes tissue factor, which triggers the coagulation cascade (1).

The coagulation system works together with platelets, leukocytes, and the complement system when endothelial cells are damaged. The coagulation and complement systems share common proteolytic pathways that enable an inflammatory response. In endothelial cells and neutrophils, C5a and C5b-9 induce tissue factor expression, initiating the extrinsic coagulation cascade. In addition, C5a induces secretion of von Willebrand factor and P-selectin, as well as increasing neutrophil adhesion in endothelial cells in culture. In addition, coagulation factors can activate the complement system at different levels (50). This interaction can be observed clinically in glomerular diseases associated with intense manifestations of TMA and antiphospholipid syndrome, including some with thrombomodulin and plasminogen variants (54).

#### 4.1.7 Circulating cells within the endothelial compartment

The endothelial compartment comprises circulating endothelial cells (CECs), endothelial cell-derived microparticles (EMPs), and endothelial progenitor cells (EPCs). After endothelial injury, it is crucial that there is a balance between CECs and EMPs (released into the bloodstream after endothelial injury)—and the capacity for endothelial repair by EPCs. The activation of EMPs is also closely related to the activation of the alternative complement pathway, which can favor the development of TMA (55). The EPCs promote angiogenesis and microvascular repair through the production of VEGF, fibroblast growth factor 2, and angiopoietin (1).

In the setting of vascular injury with endothelial dysfunction, regardless of the etiology, markers of injury/activation (circulating endothelial cells and EMPs) are at high levels, while endothelial repair markers (endothelial progenitor cells and circulating angiogenic cells) are at low levels (1).

### 4.2 Genetic markers

The endothelial response to injury also involves genetic influence. The presence or absence of adequate expression of certain genes can contribute to the occurrence of vascular lesions secondary to severe endothelial damage. In TMA, especially in aHUS, and C3 glomerulopathy, genetic sequencing studies have contributed to the identification of genetic variants involved in the complement pathways, such as: CFH, CFI, MCP/CD46, complement factor B (CFB), thrombomodulin (THBD), C3 and others (56, 57). Variants in complement genes have also been described in TMA associated with glomerular diseases including LN (58). Local CFH seems to protect the renal endothelial cell, and its absence is associated with altered cytoskeleton, altered cell metabolism, and increased proliferation (59). In addition to the complement system, variants in genes of pathways involving the renin-angiotensin system, angiogenic factors, endothelial nitric oxide synthase (eNOS) have also been reported in many studies in association with TMA lesions (60, 61). Mutations related to hereditary thrombophilia have been reported as causes of renal vascular lesions in patients without diabetes, hypertension, or a history of smoking (62). A transcriptomic analysis revealed molecular alterations in pathways related to cell adhesion, the actin cytoskeleton, angiogenesis and apoptosis of glomerular endothelial cell after podocyte injury, demonstrating that glomerular endothelial dysfunction may be a secondary event to podocyte damage, with the involvement of p53, transforming growth factor-beta 1 (TGF-β1) and TGF-α as important mediators of this process (63).

### 4.3 Histological markers

The presence of endothelial damage can be expressed by the finding of vascular lesions on renal biopsy histology. The main vascular lesions are: microangiopathy (with or without the presence of fibrin thrombi in capillaries or small vessels), arteriosclerosis, fibrosis and/or intimal thickening and arteriolar hyalinosis.

In addition to the anatomical expression of vascular involvement, it is possible to detect endothelial injury through immunohistochemistry. In individuals with such injury, there is a detectable expression of a series of factors (64–67): proteins related to the endothelial-to-mesenchymal transition (e.g., fascin-1, vimentin, and heat shock protein 47); glycocalyx-related proteins (heparan sulfate domains); endothelial adhesion molecules (e.g., vascular adhesion cell molecule-1 and platelet endothelial cell adhesion molecule-1); and angiogenic factors (e.g., VEGF receptors and AngII receptors).

#### 4.3.1 TMA in renal histology

**Figure 3:**
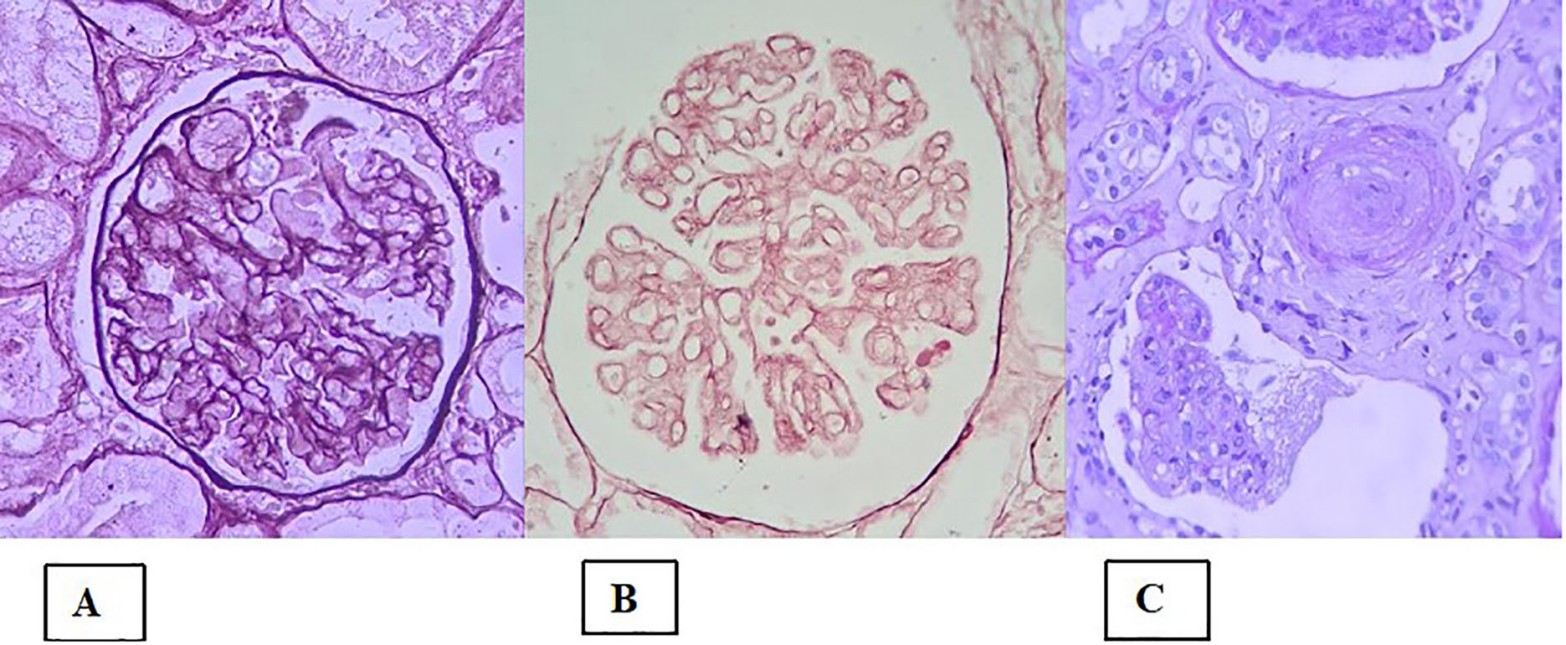
Histological lesions in the presentation of thrombotic microangiopathy. **(A)** Global mesangiolysis; **(B)** Extensive double contours of the glomerular basement membrane (two-layer appearance); **(C)** Concentric myointimal proliferation in an “onion-skin” pattern, with mucoid edema and lumen obliteration. There is also a glomerular tuft with a retracted appearance and a wrinkled basement membrane (lower portion of the image).

Classically, TMA is defined as a pathological lesion characterized by endothelial damage and the formation of microthrombi in small vessels (68). It can manifest clinically as microangiopathic hemolytic anemia, thrombocytopenia and ischemia (68). In the kidney, the acute finding of fibrin thrombus (often accompanied by fragmented red blood cells) is not always present. However, the presence of other morphological changes constitutes the aspect of microangiopathy. For example, in the active/acute phase the following lesions can be present: in glomeruli—endothelial edema, mesangiolysis (**Figure 3A**), and microaneurysms; in arterioles—endothelial/intimal edema, intramural fibrin, and myocyte necrosis; and in arteries—myxoid intimal edema and intramural fibrin. Electron microscopy (EM) shows glomerular endothelial cells with loss of fenestrations, fibrin tactoids with fragmented red blood cells and platelets, and expansion of the inner lamina. In the chronic phase, the main findings on light microscopy are as follows (68–70): double-contour sign in the glomerular capillary basement membrane (**Figure 3B**) - which appears in the EM as interposed cells with deposition of new matrix material; arteriolar hyalinosis; and thickening/fibrosis of the arterial intima, with an “onion-skin” appearance (**Figure 3C**). In the absence of a fibrin thrombus and of clinical signs of a TMA syndrome, there is still no consensus regarding how many of these other lesions must be present to confirm TMA or whether they would serve only to describe microangiopathy alone with or without thrombus. In such a situation, the diagnosis of TMA would be presumptive because other lesions are typically present in the classic form.

##### 4.3.1.1 TMA in glomerulopathies

Historically, the initial classification of TMA was based on clinical manifestation, with two main spectra: thrombotic thrombocytopenic purpura, in which there is greater neurological involvement; and HUS, in which there is greater renal involvement. Subsequently, the classification evolved toward a molecular basis of the disease (68): a disintegrin and metalloprotease with thrombospondin type one repeats, member 13 (ADAMTS13) deficiency is associated with thrombotic thrombocytopenic purpura; and HUS is classified as typical or atypical depending on whether shiga toxin is present or absent (HUS and aHUS, respectively). The discovery of dysregulation of the complement system in a significant proportion of patients with aHUS led to the classification of this subcategory as complement-mediated aHUS and contributed to the development of drugs that act to block components of the complement system (such as the C5 blocker eculizumab), leading to major improvements in the clinical outcomes in such patients (7). Concomitant with those discoveries, there have been numerous reports of TMA associated with other factors, such as autoimmune diseases, malignancy, drug use, pregnancy, malignant hypertension, glomerulopathies, and transplantation. Those cases were classified as secondary TMA, whereas those involving complement dysregulation due to genetic or acquired factors would be classified as primary TMA (68). However, in many secondary causes, including glomerulopathies, the intrinsic amplification/dysregulation of the complement pathway has been observed, which has even led to the use of complement system blockers, with a satisfactory therapeutic response in glomerular diseases, such as ANCA-associated vasculitis, in C3 glomerulopathy, in LN, and in IgAN, although it has not been specifically studied in those glomerulopathies expressing or associating with TMA (71).

The occurrence of TMA in glomerulopathies confers greater severity and a worse prognosis, with many cases quickly progressing to renal failure and the need for dialysis or kidney transplantation (68). The main autoimmune etiologies of TMA are LN, antiphospholipid syndrome, systemic sclerosis and Sjögren’s syndrome (72). Systemic lupus erythematosus is an autoimmune disease historically related to activation of the complement system, mainly from the classical pathway due to the presence of immune complexes. The occurrence of TMA in LN increases severity, lowers the therapeutic response, worsens the prognosis, and might be related to deficiency of regulatory factors in the complement pathway, leading to amplification of the pathway, culminating in the formation of a membrane attack complex, endothelial injury, and platelet aggregation (fibrin microthrombi) in the lumen of small vessels (42, 68). In IgAN, the pathogenesis of TMA is not well defined, although there is growing evidence of involvement of the complement system, based on the identification of deficiency of regulatory factors or complement activation by IgA immune complexes (71). In IgAN, TMA is associated with an unfavorable clinical outcome (2). In ANCA-associated vasculitis, endothelial injury arises mainly from products toxic to the endothelium released by neutrophils that are activated by autoantibodies (ANCAs). Several studies have demonstrated the involvement of the complement mediators, especially anaphylatoxin C5a, through activation from the initial process itself (1, 71, 73). In fact, treatment for ANCA-associated vasculitis aimed at blocking the complement system has shown good efficacy (74). Dysregulation of the complement pathway—by genetic defects, mainly related to CFH or by autoimmune diseases, mainly related to autoantibodies (such as C3, C4, and C5 nephritic factors) against components of the pathway—results in C3 glomerulopathy. Amplification of the pathway ultimately culminates in endothelial injury (71). The occurrence of TMA in patients with FSGS or minimal change disease is characterized by steroid-resistant nephrotic syndrome. Factor H deficiency has been reported in some cases (5, 75). In addition, TMA is a common finding in collapsing glomerulopathy, suggesting that endothelial injury is involved in its pathogenesis (76). There have been reports of cases of membranous nephropathy in combination with thrombotic thrombocytopenic purpura, even in patients testing positive for anti-phospholipase A2 receptor antibodies, probably due to the presence or stimulation of the development of antibodies against ADAMTS13 (6, 77). In many cases of glomerulopathy, the occurrence of TMA is not accompanied by systemic involvement with thrombocytopenia and hemolytic anemia, being a histopathological finding, which raises the hypothesis of local activation of the complement system involving regulatory factors on the endothelial surface or in other glomerular structures (42).

#### 4.3.2 Arteriolar hyalinosis in renal histology

**Figure 4:**
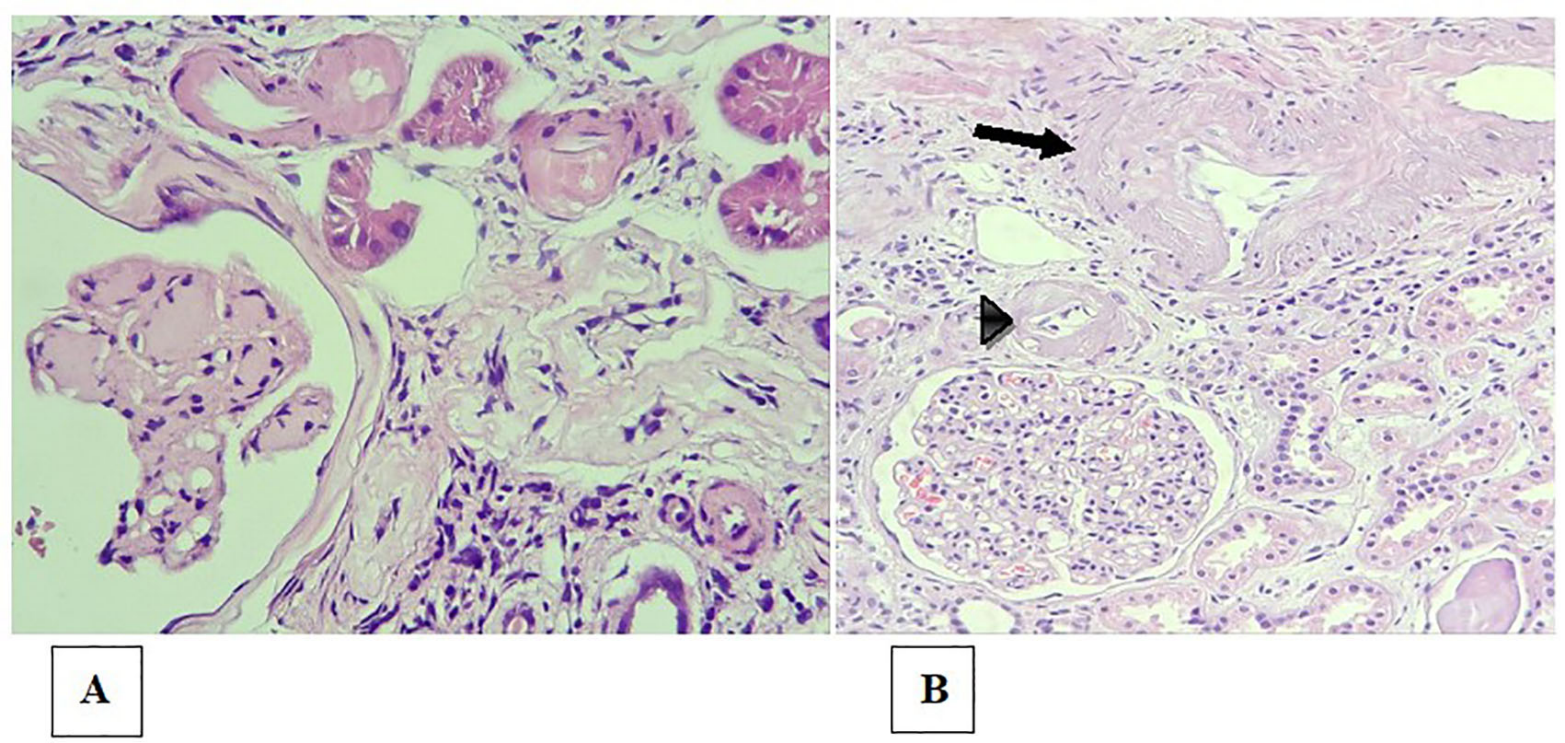
Arteriolosclerosis and renal arteriosclerosis. **(A)** Bulky, circumferential mural hyaline deposits in the arteriole walls (upper portion of the image), in a patient with diabetes and nodular glomerulosclerosis (lower left corner of the image); **(B)** Moderate intimal fibrosis in the interlobular arterial branch (arrow) and intense arteriolar hyalinosis (arrowhead).

Arteriolar hyalinosis, also known as hyaline arteriolosclerosis, is the deposition of proteinaceous material with a hyaline appearance in the subendothelial region of arterioles (78). That deposition results in thickening of the vessel wall and narrowing of the lumen, leading to ischemia (**Figure 4A**). Arteriolar hyalinosis can be observed in healthy individuals as they age. However, it occurs earlier in patients with hypertension or diabetes mellitus.

##### 4.3.2.1 Arteriolar hyalinosis in glomerulopathies

Arteriolar hyalinosis is well described as a predictor of renal outcome in kidney transplant recipients, diabetes, and hypertension patients (79, 80). In patients with glomerulopathies, arteriolar hyalinosis can be associated with hypertension concomitant with glomerular disease, although its clinical significance and role in the pathogenesis of glomerular disease, especially in the presence of other vascular lesions such as TMA, still need to be clarified. In IgAN, for example, arteriolar hyalinosis is an independent risk factor for an unfavorable renal prognosis (81). Arteriolar deposition of C4d in biopsies of patients with IgAN is associated with glomerular C4d deposition and both are associated with the progression of kidney disease (82). In steroid-resistant FSGS, arteriolar hyalinosis can hinder the treatment, because the use of cyclosporine can worsen renal function in such cases (83). In renal biopsy studies, a finding of arteriolar hyalinosis is a risk factor for the progression of renal disease (84).

#### 4.3.3 Arteriosclerosis in renal histology

Arteriosclerosis is characterized by thickening of the intimal layer by smooth muscle fibers or fibroblasts, collagen fibers and the fundamental connective tissue that causes narrowing of the vessel lumen (**Figure 4B**). It does not have the homogeneous appearance characteristic of arteriolar hyalinosis, with which it should not be confused (78). It is also commonly found in elderly individuals, individuals with hypertension, and individuals with diabetes.

##### 4.3.3.1 Arteriosclerosis in glomerulopathies

Arteriosclerosis has been well characterized in patients with arterial hypertension or diabetes mellitus (85, 86). In the context of kidney transplantation, a finding of arteriosclerosis is correlated with shorter graft survival in all cases, including those in which the donor kidney is from a donor with uncontrolled hypertension, representing a relevant variable in the Banff classification criteria (87, 88). In patients with LN, the use of the Banff score to evaluate vascular lesions showed that the prevalence of renal arteriosclerosis is accelerated by two decades in such patients, which constitutes an early cardiovascular risk factor (89). Severe arteriosclerosis is associated with shorter renal survival in patients with crescentic glomerulonephritis (90). In other glomerulopathies, the meaning of a finding of arteriosclerosis on renal histology is poorly understood.

## 5 Endothelial dysfunction in glomerular diseases

There are some glomerular diseases in which the pathogenesis of vascular lesions is partially understood, such as diabetic kidney disease and hypertensive nephrosclerosis. In other situations, especially in primary glomerulopathies, the pathogenesis of vascular lesions is not yet well defined, with potential mechanisms being the activation of the complement system and dysregulation of pathways involving podocyte–endothelium and tubule/interstitium–endothelium interactions.

### 5.1 Potential mechanisms of endothelial involvement in glomerulopathies

The mechanisms of endothelial dysfunction in glomerular diseases can involve direct or indirect damage with endothelial cell activation or deficiency of factors that regulate or protect the endothelium. **Table 1** summarizes the potential mechanisms.

**Table 1:** Potential mechanisms of endothelial dysfunction in glomerulopathies.

Glomerular disease	Potential mechanisms of endothelial dysfunction
Lupus nephritis	Immune complex deposition causes endothelial cell damage. There is increased expression of adhesion molecules; increased secretion of interleukins (IL-6 and IL-8); increased levels of tumor necrosis factor alpha, chemokine CCL2, and nitric oxide; inhibition of angiogenesis; activation of complement pathways with direct and indirect endothelial injury; and imbalance of regulatory and activating factors of endothelial cells due to a functional defect mediated by interferon I, making them susceptible to apoptosis. There can be production of specific antibodies that act on the endothelial cell surface (1, 15, 91).
ANCA-associated vasculitis	Neutrophils activated by antineutrophil cytoplasmic antibodies adhere to the endothelial cell, leading to activation of the endothelium and increased vascular permeability. Activated neutrophils release toxic granules, causing direct endothelial cell injury and fibrinoid necrosis through an increase in reactive oxygen species, proteases, and neutrophil extracellular traps. There is activation of the alternative complement pathway with amplification of inflammation through the recruitment of anaphylatoxins, especially C5a, leading to greater endothelial dysfunction (14, 73, 92).
IgA nephropathy	Deposition of IgA1 in the mesangium increases the local inflammatory response, leading to endothelial damage due to mesangium–endothelium interaction with increased nitric oxide synthase. The high affinity of IgA1 for the glomerular endothelium, especially in endocapillary proliferative forms with complex immune deposits, leads to the production of cytokines and adhesion molecules, as well as endothelial barrier dysfunction and loss of glycocalyx. There is activation of the complement system through the lectin pathway and deficiencies in complement regulatory factors, with consequent endothelial damage. There is also activation of the coagulation cascade from endothelial damage favoring local microangiopathy. Elevation of soluble fms-like tyrosine kinase-1 and inhibition of VEGF cause endothelial injury, a reduction in angiogenesis, and the development of anti-endothelial cell antibodies (16, 93, 94).
Focal segmental glomerulosclerosis	Podocyte injury promotes dysregulation in the podocyte–glomerular endothelium interaction mainly by reducing VEGF production, which results in endothelial damage and impaired angiogenesis. There is increased expression of the endothelin type A receptor in the endothelial cell, which induces endothelial oxidative stress (95, 96).
Membranous nephropathy	Probable endothelial cell apoptosis results from deposition of immune complexes in the glomerular capillary wall. There are low levels of plasma VEGF and urinary VEGF excretion, representing a defect in endothelial function, compromising angiogenesis (21, 67).
Membranoproliferative glomerulonephritis and C3 glomerulopathy	Dysregulation of the alternative complement pathway by antibodies, immunoglobulins, toxins or acquired intrinsic defects, among others. The change can occur more as a result of the activation of C3b receptors in the glomerular endothelium than as a result of activation of the membrane attack complex (10, 97)..
Diabetic kidney disease	Hyperglycemia induces glomerular endothelial cell apoptosis, alteration of the glycocalyx, with reduced heparan sulfate synthesis. It also interferes with VEGF receptors expressed by the podocyte, contributing to endothelial cell dysfunction and dysfunctional endothelium–podocyte interaction, as well as stimulating the change from the endothelial to the mesenchymal phenotype, thus promoting renal fibrosis (1, 12, 98).
Hypertensive nephrosclerosis	Increased blood pressure induces mechanical stress on the vessel wall, causing vascular injury and activation of the intimal thickening process, with or without hyalinosis of the arterioles and small vessels, including the glomerular capillaries. Activation of the renin–angiotensin system with increased angiotensin II promotes vasoconstriction, inflammation, and fibrosis. There is also a loss of renal autoregulation, resulting from vascular injury, and narrowing of the vessel lumen. Arteriosclerosis induces ischemia, which amplifies endothelial damage. Complement activation with endothelial dysregulation culminating in thrombotic microangiopathy occurs mainly in cases of accelerated malignant hypertension (13, 99, 100).

IgA, immunoglobulin A; VEGF, vascular endothelial growth factor.

In glomerulopathies, the pathogenesis of vascular injury is described as being mainly related to damage to the glomerular endothelium, although the approach to the involvement of arterioles and small vessels has not been well elucidated. TMA, regardless of etiology, has been linked to dysregulation of the complement system (local or systemic). However, in some glomerulopathies, even with dysregulation of the pathway, TMA does not develop, whereas it does develop in other glomerulopathies without clear evidence of complement system dysregulation. Further studies are needed in order to understand whether there are specific pathways or endothelial regulatory factor deficiencies that are related to the development of TMA in glomerular diseases. Regarding arteriosclerosis and arteriolar hyalinosis, the hypothesis that arterial hypertension and age are involved has been raised. However, some studies have shown that these types of vascular injury occur in some glomerulopathies even in the absence of hypertension and of advanced age (13, 101). The potential mechanisms involved in this situation are not clear and might be related to initial damage to the glomerular endothelium or podocyte, with consequent interaction between the tubules and the interstitium, together with a defect in renal autoregulation, as well as to factors such as hyperuricemia, elevated cholesterol, and dysregulation of complement factors (81, 102).

### 5.2 The endothelium as a therapeutic target in glomerulopathies

The endothelial cell and its mediators have always been a target of study for the development of treatments for chronic kidney disease, including glomerulopathy. The use of a renin–angiotensin–aldosterone system blocker was perhaps the first treatment for which there was robust evidence of an ability to control the progression of kidney disease and proteinuria, which made it the standard treatment for many glomerulopathies, such as IgA nephropathy and diabetic nephropathy, and for chronic kidney disease itself (103, 104). Concomitant to the discoveries related to angiotensin in numerous studies, the effects of ET-1 and the benefits of selective endothelin A receptor blockade were also described, mainly in the reduction of renal progression (105). Recently, the recovery and refinement of knowledge regarding these mechanisms has led to the development of new, more selective drugs for blocking the receptors of angiotensin, endothelin, and aldosterone, alone or in combination (46, 47, 106). In addition, the combination of these drugs with a sodium-glucose cotransporter 2 (SGLT2) inhibitor has shown clinical benefit, which also demonstrates a role for SGLT2 inhibitors in endothelial activity (48, 107). After the advent of SGLT2 inhibitors, which have provided great benefit in reducing proteinuria and progression of kidney disease, many studies have focused on the mechanisms of those effects. In fact, recent evidence shows that SGLT2 inhibitors achieve their antioxidant and anti-inflammatory effects by reducing the expression of endothelial adhesion molecules, thus preventing endothelium–leukocyte interaction (108). In relation to TMA, most studies have focused on blocking the complement system, taking into account the change in clinical evolution in patients with aHUS treated with C5 blockade. The finding of complement protein deposits, either alone or in combination with immune complexes and local inflammation, has led to the study of complement blockers as treatments for glomerulopathies. In C3 glomerulopathy with or without microangiopathy, the fact that the pathogenesis involves dysregulation of the alternative pathway makes the indication for blockade more specific (103). In other glomerular diseases, complement blockade appears to play a role in reducing inflammation. In ANCA-associated vasculitis, for example, treatment with complement blockade has been shown to provide a clinical benefit, especially in controlling the action of anaphylatoxin, through blockade of the C5a receptor (74). In the presence of microangiopathy accompanying glomerulopathies, despite the reference to TMA mechanisms in aHUS, there is little evidence to support the use of complement blocking drugs, although some studies and case reports have shown therapeutic potential in certain situations. **Table 2** summarizes the studies that have associated complement blockade as a specific treatment for TMA in glomerulopathies.

**Table 2:** Complement blockade in glomerulopathies with TMA.

Glomerulopathy with TMA	Genetic Variant	Complement Blocker	Renal Clinical Response
LN			
de Holanda MI, et al., 2017 (2 cases) (109)	CFHR1/CFHR3	Eculizumab	Improvement
Raufi AG, et al., 2016 (110)	Absent	Eculizumab	Improvement
El-Husseini A, et al., 2015 (111)	Not performed	Eculizumab	Improvement
Coppo R, et al., 2015 (112)	Absent	Eculizumab	Improvement
Bermea RS, et al., 2016 (113)	Not performed	Eculizumab	No improvement
Torres EA, et al., 2021 (114)	CFHR1-3	Eculizumab	Partial improvement (incremental dialysis).
Kim MJ, et al., 2021 (115)	C3 mutation	Eculizumab	Improvement
Kello N, et al., 2019 (116) (considering 3 cases of LN without APS)	Not performed	Eculizumab	1 case: improvement 2 cases: no improvement
Park MH, et al., 2018 (54) (considering 7 cases of LN without APS and renal transplant)	CFH in patient A Absent in patients B, C, and D Not performed in patient E Thrombomodulin, Plasminogen and CFH in patient F CFHR1-CFHR3, Plasminogen, and MCP in patient G	Eculizumab	Improvement in A, B, D, and G No improvement in C, E, and F
Ono M, et al., 2018 (117)	Not performed	Eculizumab	No improvement
Cavero T, et al., 2017 (118) (considering 3 cases)	Absent in 2 cases/Not performed in 1 case	Eculizumab	No improvement in 2 cases and partial improvement in 1 case
Smith J, et al., 2024 (119)	CFH	Eculizumab	Improvement
ANCA vasculitis			
Cao M, et al., 2017 (120)	CFH;	Eculizumab	Improvement
Cavero T, et al., 2017 (118) (considering 2 cases)	Absent/CFHR1	Eculizumab	Partial improvement/No improvement
IgAN			
Patel DM, et al., 2021 (121)	Not performed	Eculizumab	Improvement
Matsumura D, et al., 2016 (122)	Not performed	Eculizumab	No improvement
Nakamura H, et al., 2018 (123)	CFH	Eculizumab	Partial improvement
C3G			
Chabannes M, et al., 2023 (124) (Considering 10 cases)	CFH (3 cases)/Absent (5 cases)/Not performed (2 cases)	Eculizumab	Improvement in 6 cases (2 cases with CFH mutation)
Ravindran A, et al., 2022 (125) (Considering 1 case treated with eculizumab)	Absent	Eculizumab	No improvement
Osawa K, et al., 2023 (126)	CFI	Eculizumab/Ravulizumab	Improvement

LN, Lupus Nephritis; ANCA, Antineutrophilic cytoplasmic antibody; IgAN, IgA Nephropathy; C3G, C3 Glomerulopathy; APS, antiphospholipid syndrome.

Most case reports of glomerular disease with TMA and treatment with complement blockade include LN, ANCA vasculitis, IgAN and C3 glomerulopathy. With regard to FSGS and membranous nephropathy, published case reports on the use of complement blockers mainly involve the occurrence of TMA after kidney transplantation (127, 128). Minimal Change Disease with TMA has, to date, no published case of specific treatment with complement blockade.

In LN with TMA, a systematic review suggests that the use of eculizumab may be beneficial, especially in refractory situations, but some of the included studies showed an association of SLE with antiphospholipid syndrome (APS), which represents another aspect of the disease (129). **Tables 2** show cases of LN with TMA and without APS, which demonstrate great variability in the response to eculizumab, some without testing for genetic variants, which makes it difficult to define a clinical decision on the use of complement blockade in this condition. In situations where ADAMTS13 activity is normal and antiphospholipid antibody is negative, complement system analysis is recommended, since many cases are related to complement-mediated TMA that is resistant to conventional treatment, with eculizumab being a considerable therapeutic option, although dosage and duration are not well defined (130).

In TMA-ANCA vasculitis and in the TMA-IgAN, **Table 2** shows cases with variable results, and it is not possible to conclude recommendations due to the lack of evidence considering TMA association, although for ANCA-vasculitis, in general, C5a receptor blockade has shown good efficacy (74).

In C3G with TMA, a case series revealed that renal survival was significantly improved in patients treated with eculizumab, and it is reasonable to consider the complement blocker in this situation if no response is observed after conventional treatment (124).

In hypertensive nephrosclerosis, the occurrence of TMA is more common in situations of malignant hypertension. Recently, the discovery of a higher proportion of pathogenic genetic variants in these cases has raised the suspicion that many are complement-mediated TMA presenting with severe hypertension (131). A registry analysis evaluated the use of eculizumab in patients with TMA/aHUS and malignant hypertension, suggesting that it may be an effective alternative (132). The decision to treat with complement blockers depends on classifying the condition as complement-mediated TMA with severe hypertension, rather than TMA secondary to hypertension, which is quite difficult in clinical practice. Some authors suggest considering cardiac hypertrophy and the predominance of arteriolar lesions as characteristics more related to hypertension-associated TMA, while increased glomerular involvement and the lack of response to aggressive antihypertensive therapy are more related to complement-mediated TMA with severe hypertension (133).

Diabetic kidney disease with TMA has, to date, no published case of specific treatment with complement blockade.

It is known that the mechanisms of involvement of the complement system vary between diseases, sometimes being non-specific and in others playing a central role in the pathogenesis, justifying the difference in therapeutic response. Unfortunately, there is no exact test and no solid evidence that would justify the widespread use of complement blockade in glomerulopathy with TMA (134). Low levels of complement proteins, such as C3, may not be present in many cases of system dysregulation (135). Although the involvement of complement can be evaluated with detection of autoantibodies to complement factors and by functional assays, such as soluble C5b-9 and tissue deposition of C5b-9, so far only genetic tests have been able to distinguish complement dysregulation from overactivation/amplification (135, 136). However, it is known that the genetic test is not available to everyone, the absence of detectable genetic mutation does not rule out complement-mediated TMA and that the cost of the drug is high. On the other hand, the worsening clinical evolution despite conventional treatment of the glomerulopathy has become increasingly worrying, which makes it urgent to define the situations in which the use of complement blockers can be beneficial. Some authors advocate the use of complement blockade in secondary TMA to control transient overactivation of the complement system and reduce endothelial damage when there is no response to standard treatment and, once the initial condition has resolved, the complement blocker can be discontinued (137). Some studies have shown that in the absence of pathogenic variants in complement genes, the risk of relapse after discontinuation is low, although glomerulopathies has low representative in these studies (118). Conversely, if pathogenic variants are detected, the case may be primary TMA and the glomerulopathy may have occurred as a trigger. In this situation, the use of complement blockers should be extended (137). It seems reasonable to use complement blockers when dysregulation of the complement system is detected by the presence of a genetic variant as a determining factor in pathogenesis and when this is documented by accumulated scientific evidence, as is the case with C3G.

Therefore, the decision to start a complement blocker in glomerulopathies with TMA should be based on clinical evolution, response to conventional treatment, evidence of complement involvement and the results of genetic tests. More importantly, inclusion in randomized clinical trials will allow for a more precise response to this controversy. In addition, microangiopathy lesions in glomerular diseases may be related to defects in endothelial cell regulators, dysregulation of the local coagulation system, angiogenesis defects and other pathways besides the complement system, as already mentioned in this article. For example, there are some studies that show a better clinical response with the use of anticoagulation in cases of TMA and LN + APS, which is a recommendation of the latest KDIGO update (130, 138). With regard to endothelial cell regulators, there is emerging evidence that glycocalyx could be a therapeutic target in TMA (139).

In conclusion, glomerular disease can cause damage to the glomerular endothelial cell in many structures and pathways (complement system; angiogenesis-related factors; renin-angiotensin system; endothelin complex; coagulation cascade; interaction between these pathways; interactions between glomerular structures and interstitial structures), which may represent different potential therapeutic targets to explore in the case of vascular damage associated with glomerular diseases, in particular TMA.

## 6 Conclusion

Endothelial dysfunction and the presence of vascular disorders such as TMA, arteriolar hyalinosis, and arteriosclerosis are associated with a more severe clinical course and a worse renal prognosis in glomerulopathies. In glomerular diseases, the mechanisms of endothelial dysfunction can involve direct or indirect damage with endothelial cell activation or deficiencies of factors that regulate or protect the endothelium. Blocking the RAS and endothelin are therapeutic strategies that act on mechanisms related to endothelial cells and have provided clinical benefit in reducing proteinuria and slowing the progression of kidney disease. The use of complement blockade has increased in diseases with clear evidence of impairment of the complement system and consequent endothelial damage, such as TMA. However, not all glomerular diseases present concomitant vascular damage, even in the presence of endothelial dysfunction. That raises the hypothesis that a potential intrinsic defect in endothelial regulation/protection is involved. A greater understanding of the pathogenesis of vascular injury could lead to specific therapeutic advances in this most severe manifestation of glomerular disease.
